# ﻿Three new species of *Trimmatothelopsis* (Acarosporales, Acarosporaceae) from southwestern North America

**DOI:** 10.3897/mycokeys.99.102965

**Published:** 2023-10-12

**Authors:** Kerry Knudsen, Jana Kocourková, Eva Hodková, Jason Dart, Alejandro Huereca, Jiří Malíček

**Affiliations:** 1 Czech University of Life Sciences Prague, Faculty of Environmental Sciences, Department of Ecology, Kamýcká 129, Praha - Suchdol, 165 00, Czech Republic Czech University of Life Sciences Praha Praha - Suchdol Czech Republic; 2 Althouse and Meade, Inc. 1650 Ramada Drive, suite 180, Paso Robles, CA 93446, USA Althouse and Meade, Inc. Paso Robles United States of America; 3 Robert F. Hoover Herbarium, Biological Sciences Department, California Polytechnic Institute, San Luis Obispo, CA, 93407, USA Biological Sciences Department, California Polytechnic Institute San Luis Obispo United States of America; 4 University of Alberta, Department of Biological Sciences CW405, Edmonton, AB T6G 2R3, USA University of Alberta Edmonton Canada; 5 The Czech Academy of Sciences, Institute of Botany, Zámek 1, 252 43 Průhonice, Czech Republic The Czech Academy of Sciences, Institute of Botan Průhonice Czech Republic

**Keywords:** Ascus stains, California, Chihuahuan Desert, conidia, conidiogenous cells, Mexico, New Mexico, pycnidia, rare species

## Abstract

The discovery and study of three new species of *Trimmatothelopsis* from Southwestern North America, *T.californica*, *T.mexicana*, and *T.novomexicana*, adds not only to the diversity of the genus and family but generated new insights into the occurrence of two ascus types in the genus and the variety of conidiogenous cells and conidia. *Trimmatothelopsis* now includes 15 species with a mainly Holarctic distribution (Asia, Europe, North America) and one species in Australia. A key is supplied to the genus. An overview of the genus *Trimmatothelopsis* is given, including differentiation from other genera of Acarosporaceae. The monotypic genus *Thelocarpella* is considered to be a synonym of *Trimmatothelopsis*. The new combination *Trimmatothelopsiswirthii* is proposed. The ascus type is shown to be variable in the genus with species with two types being intermixed with each other in our phylogeny.

## ﻿Introduction

There are estimated to be approximately 416 described species of Acarosporaceae worldwide (Knudsen et al. 2023). Before these new discoveries in this paper, the genus *Trimmatothelopsis* included 11 crustose lichens, which occur on calcareous and non-calcareous rocks or in soil crusts in Asia, Australia, Europe, and North America (Knudsen and Lendemer 2016; Knudsen et al. 2021a). In southwestern North America the genus was only known from California and Nevada. For a history of the genus see Knudsen et al. 2021a.

The main form of the thallus in *Trimmatothelopsis* is areoles or squamules dispersed and/or with congregations of thalline units but not forming an areolate crust. *Trimmatothelopsisamericana*, for instance differs in having carbonized lecideoid apothecia with its algal layer occurring in a biofilm at its base. The terricolous species, *T.benedarensis*, *T.rhizobola*, and *T.terricola* have a hypothallus of well-developed rhizohyphae with the two latter species having rhizohyphae in root-like bundles. In the genus, the areoles or squamules are either pale with a reddish brown circle around the apothecia (some specimens of *T.dispersa* and *T.schorica*), or light or dark brown. The apothecia of *Trimmatothelopsis* have a disc usually 0.5 mm or less in width (Knudsen and Lendemer 2016). In all species the hymenium is 150–350 µm high, globose, and widest at the equator. The paraphyses are thin, 1–2 µm wide. The asci contain 200–300 ascospores or more. The ascospores are usually ellipsoid and not longer than 5 µm, except in *T.schorica* in which they are spherical, 7–10(–12) µm, or broadly ellipsoid 7–9 × 5–7 µm.

The asci in the genus are functionally unitunicate but some variation is found among the species. While most of the species of the genus have an IKI- *Acarospora*-type ascus stain (Hafellner 1993), *T.americana* and *T.gordensis* have an ascus with IKI+ light blue tholus and space between the inner and outer wall of the ascus with a darker blue area in the upper layers of the tholus. *Trimmatothelopsismontana* has a blue ascus stain but in the type specimen no darker layer was observed in the upper layers of the tholus.

The subhymenium is either IKI+ blue and euamyloid or hemiamyloid, blue turning red. The hypothecium is usually narrow 10–40 μm continuous with a narrow parathecium of usually the same width that merges into the cortex. The algal layer is usually continuous and not interrupted, extending down the sides of the apothecia. The medulla is usually ca. 200 μm thick. No secondary metabolites have been detected with thin-layer chromatography.

Conidia were reported from eight of the eleven species (*T.americana*, *T.benedarensis*, *T.gordensis*, *T.oreophila*, *T.rhizobola*, *T.schorica*, *T.terricola*, *T.versipellis*) (Navarro-Rosinés et al. 1999; Knudsen et al. 2011; Gueidan et al. 2014; Knudsen and Lendemer 2016; Knudsen et al. 2021a). The long conidia distinguish *Trimmatothelopsis* from the other genera of Acarosporaceae, except the monotypic genus *Lithoglypha* from South Africa, which so far, we have failed to sequence and has long conidia 3.5–7.5 × 0.8 μm.

The IKI+ blue ascus stain has some similarity to the ascus stains in the genera *Timdalia* and *Pleopsidium* but neither have a darker blue area in the upper layers of the tholus (Hafellner 1993; Hafellner and Türk 2001). Both genera are monophyletic with *Timdalia* also differing in producing psoromic acid and *Pleopsidium* in having yellow thalli (Westberg et al. 2015). Because of the high hymenium the species of *Trimmatothelopsis* are easily confused with the monophyletic genus *Myriospora* which has an *Acarospora*-type ascus stain (Westberg et al. 2011). In determining specimens, one must first determine the appropriate genus. The high hymenium and globose apothecia easily separate *Trimmatothelopsis* species from most of the species of the non-monophyletic *Sarcogyne* and *Acarospora* groups, both which have *Acarospora*-type ascus stains.

Our objective is the taxonomic and phylogenetic study of the rich diversity of Acarosporaceae in southwestern North America where occur 93 described species and where new taxa are still being discovered (Knudsen et al. 2021b; Knudsen et al. 2023). This study of diversity is laying the foundation for phylogenomic work which we have begun with our current study of the *Acarosporastrigata* group and possible hybridization and introgression in the evolution of the family.

## ﻿Material and methods

### ﻿Herbarium study

We studied our recent collections and specimens in SBBG (UCR lichen herbarium transferred to SBBG in 2022 and 2023), at OBI, and in the private herbarium of Jana Kocourková and Kerry Knudsen (hb. K&K). This continues our study of the species included in this genus since 2011 (Knudsen et al. 2011; Knudsen and Lendemer 2016. Knudsen et al. 2021a). The morphology of specimens was examined with dissecting microscopes. At 1000× with compound microscopes the anatomy of hand sections was examined and measured in water. Ascospore and conidia measurements of species are indicated as (min–)(*x̄* − SD)–*x̄*–(*x̄* + SD)(–max), where ‘min’ and ‘max’ are the extreme values observed, *x̄* the arithmetic mean and SD the corresponding standard deviation. They are followed by the number of measurements (n); the length/breadth ratio of ascospores is indicated as l/b and given as *x̄* the arithmetic mean value. The amyloid reaction of the hymenial gel and subhymenium was tested with fresh undiluted IKI (Merck’s Lugol for the gram staining method, Sigma-Aldrich 1.09261) (see protocol in Knudsen and Kocourková 2018). The ascus stain was studied in IKI (Hafellner 1993). Thin-layer chromatography (TLC) in solvents A, B’, C was performed to identify secondary metabolites (Orange et al. 2001). On completion of the study holotype, isotype and paratype material was placed in BYU-C, SBBG and PRM.

### ﻿Imaging

Macrophotographs were taken with the digital camera Olympus DP74 mounted on Olympus SZX 16 stereomicroscope using PROMICRA QuickPHOTO CAMERA 3.3 software and stacked using Olympus DeepFocus 3.5 module for increasing the depth of field. Microphotographs were taken with a digital camera Olympus DP74 mounted on an Olympus BX51 light microscope fitted with Nomarski interference contrast and using PROMICRA QuickPHOTO CAMERA 3.3 software. The figure plates were processed with the module Figure Maker fitted to the same software.

### ﻿DNA extraction, PCR amplification and sequencing

DNA was extracted from 12 dried herbarium specimens via (Suppl. material 1) the Invisorb Spin Plant Mini Kit, according to the manufacturer’s protocol with slight modifications (i.e. eluted in 50 μL of DNA, instead of 100 μL, and incubated in buffer for 15 minutes before final centrifuging). Total extracted DNA was stored at -20 °C. The quality and yield of DNA isolated was checked on a 1% agarose gel and DNA concentration and purity were then measured precisely using a UVS‐99 spectrophotometer (ACTGene). The selected markers for this study were the internal transcribed spacer (ITS; White et al. 1990), the large subunit of the nuclear ribosomal DNA (nrLSU; Vilgalys and Hester 1990), and the small subunit of the mitochondrial ribosomal DNA (mtSSU; Zoller et al. 1999). The ITS, nrLSU, and mtSSU regions were amplified via polymerase chain reaction (PCR).

Each reaction contained 1 μL (20–25 ng) of extracted genomic DNA, 10 μL of 2x MyTaq Red DNA Polymerase (Bioline), 8.2 μL of water, 0.4 μM of forward/reverse primer (10 μM) for a total reaction volume of 20 μl. Conditions for nrITS, mtSSU nrDNA: initial denaturation 95 °C for 5 min, followed by five cycles (95 °C for 33 s, 56 °C for 30 s, and 72 °C for 30 s), then ten cycles (95 °C for 30 s, 54 °C for 30 s, and 72 °C for 30 s), and twenty cycles (95 °C for 30 s, 50 °C for 30 s, and 72 °C for 30 s) with a final extension 72 °C for 15 min. Conditions for the nLSU: initial denaturation 95 °C for 1 min, followed by five cycles (95 °C for 30 s, 55 °C for 30 s, and 72 °C for 60 s) and finally 30 cycles (95 °C for 30 s, 52 °C for 30 s, and 72 °C for 60 s), with a final extension 72 °C for 10 min. Before sequencing, the PCR products were purified using the enzymatic method ExoSap-ITTM Express Reagent provided by Thermo Fisher (Scientific, Inc.) according to the manufacturer’s protocol. PCR products were run on a 1.0% agarose gel via electrophoresis and stained with ethidium bromide for 20 min. Purified PCR products, water, and forward primer (8 μL in total volume) were sequenced by BIOCEV, Vestec, Czech Republic.

### ﻿Sequence alignment and phylogenetic analysis

Sequences were checked against the UNITE and NCBI databases for contamination. All newly generated sequences were deposited in GenBank (Suppl. material 1). The sequences were proofread and concatenated manually into a single data set using SEQUENCHER version 5.4.6 (GeneCodes). Sequences were aligned using the multiple sequence alignment online service MAFFT version 7 with ‘G-INS-1’ strategy (Katoh and Toh 2008). Indels longer than 1 bp were coded by the simple gap coding method (Simmons and Ochoterena 2000) as implemented in SEQSTATE 1.4.1 (Müller 2005). A partition homogeneity test (ILD) with heuristic search was performed under one thousand replicates between the ITS, nLSU, and mtSSU sequences by PAUP* version 4.0a169 (Swofford 2002) to determine whether the partitions were homogeneous for test of congruence. The final alignments are accessible at TreeBASE database (https://treebase.org/) under submission ID 29625. For phylogenetic analyses two trees were generated (i.e. ITS + mtSSU + nLSU and only mtSSU data sets), the GTR+I model was selected as the best-fitting model of nucleotide substitution based on the Akaike Information Criterion using JMODELTEST 2.1.10 for each gene (Darriba et al. 2012). Phylogenetic trees were constructed using MRBAYES 3.2.2 (Ronquist and Huelsenbeck 2003). Input data was formatted for MRBAYES via the FABOX (Villesen 2007) with slightly modification (i.e. analyses were executed under the GTR+GAMMA nucleotide substitution model). Three replicate analyses with four chains each were computed 30,000,000 generations, sampling every 1000^th^ generation. After this number of runs, the average standard deviation of split frequencies reached a value lower than 0.01, indicating that convergence was reached. The data were additionally analyzed using maximum likelihood (ML) method. Tree searches for ML analyses were executed under the GTR+GAMMA nucleotide substitution model (general time reversible substitution model with a gamma model of rate heterogeneity) in RAxML v.8.2.10 (Stamatakis 2014). The Bayesian inference tree with posterior probabilities and ML phylogenetic tree with 1000 replicates were visualized using Fig-TREE v1.4.4 (Rambaut 2012). Sequences of *Pycnorasorophora* was the outgroup and five of the six recognized genera of the Acarosporaceae [three monophyletic genera (*Myriospora*, *Pleopsidium*, *Timdalia*) and selected specimens representing the non-monophyletic *Acarospora* and *Sarcogyne* groups] were used to recover the monophyletic *Trimmatothelopsis* clade. The *Trimmatothelopsis* clade contained all available sequences of the species in the genus. The results recover the same results as a large family tree in Knudsen et al. (2023).

**Figure 1.**
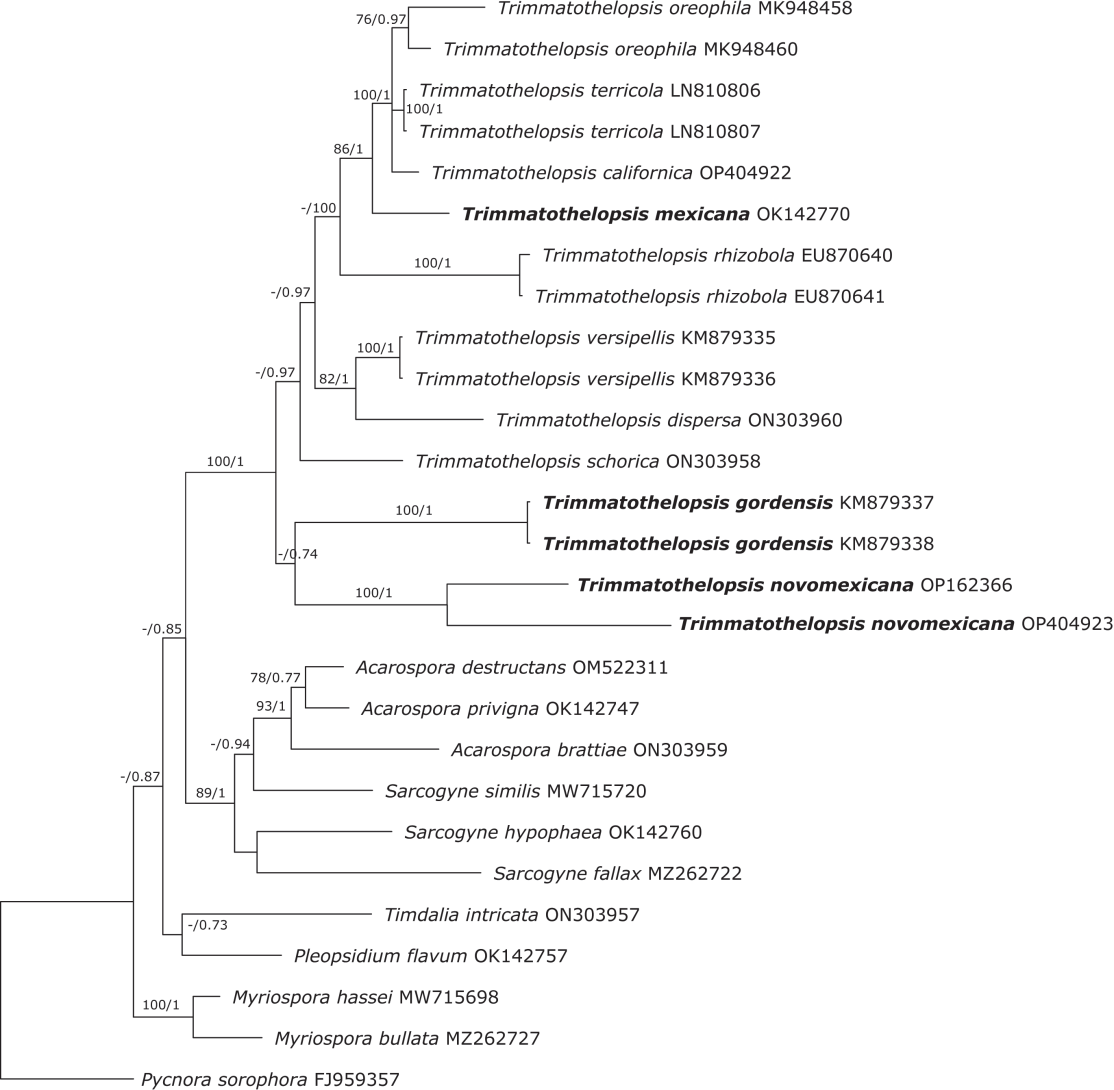
Bayesian inference tree obtained by phylogenetic analysis using a combined data set of ITS, mtSSU and nLSU sequences of 27 members of Acarosporaceae. Bayesian posterior probability (BPP ≥ 0.95) and maximum likelihood bootstrap values (ML ≥ 70%) are indicated above branches (BPP/ML). *Pycnorasorophora* was used as outgroup. In bold are species with IKI+ blue asci. The remaining species of *Trimmathelopsis* not in bold have IKI- asci.

The final alignment contained 1834 concatenated characters, consisting of 1–436 (ITS), 437–1084 (mtSSU), 1068–1834 (nLSU) nucleotide positions. Of these characters, 1338 were constant, 179 were variable and parsimony-uninformative and 317 were parsimony- informative. The topology of the ML tree confirmed the tree topology obtained from the Bayesian Inference and, therefore, only the Bayes tree is presented (Fig. 1). The MCMC analysis of the three concatenated genes was run for 30,000,000 generations, resulting in trees. The alignment contained a total of 538 unique site patterns. The analyses identified a well-supported *Trimmatothelopsis* clade with 100 percent bootstrap support (BPP) in the combined data set (Fig. 1). The new species were nested within the *Trimmatothelopsis* clade. Species relationships within the clades are resolved, and relationships amongst all clades were resolved with strong confidence.

## ﻿Results and discussion

*Trimmatothelopsiscalifornica* is most closely related to *T.oreophila* and *T.terricola* (Fig. 1). These three species are frequent in California and have an *Acarospora*-type ascus with IKI- tholus and space between inner and outer wall of the ascus. *Trimmatothelopsiscalifornica* was originally reported from California as *Myriosporascabrida* (Knudsen 2005, 2007). The species differs from all other species in *Trimmatothelopsis* in having the shortest conidia: (1.8–)2.0–2.26–2.5(–2.8) × (0.8–)0.82–0.98–1.1(–1.3) µm, l/b 2.3. In addition, it has wide discs (up to 0.5 mm wide), only surpassed by *T.dispersa* (up to 0.7 mm wide). *Trimmatothelopsismexicana* is sister to this clade (Fig. 1). It has a dark blue stain in the upper layers of the tholus and differs from other species in the genus in having long conidiogenous cells 21.0–32.0 × 1.9–2.8 µm and the longest conidia in the genus (4.1–)5.0–8.2–11.4(–13.9) × (0.9–)1.0–1.18–1.4(–1.5) µm (n = 20), l/b 7.

*Trimmatothelopsisnovomexicana* was recovered as sister to *T.gordensis* (Fig. 1). It differs from *T.gordensis* in having an areolate thallus.

In the phylogeny (Fig. 1), the species with an IKI+ ascus stain are recovered intermixed with species with an *Acarospora*-type ascus, suggesting that the variation in the amyloidity of the ascus is of limited phylogenetic importance.

The three new species of *Trimmatothelopsis* described here and the placement of *Trimmatothelopsiswirthii* (C. Roux) K. Knudsen & Kocourk. in the genus, brings the total number of species to fifteen.

### ﻿Taxonomy

#### ﻿ Trimmatothelopsis californica

Taxon classificationFungiAcarosporalesAcarosporaceae

K. Knudsen, Dart, Kocourk. & Hodková sp. nov.

CD5744ED-E5FF-5E4B-A47D-4FCE33229CC1

: MB845664

Fig. 2

##### Type.

U.S.A. Monterey Co.: Cholame Hills, along Cholame Creek, annual grassland, 35.8318; –120.3573, alt. 390 m, on granite, 12 Feb. 2016, Jason Dart 577 (holotype-BYU-C, isotype-OBI).

**Figure 2.**
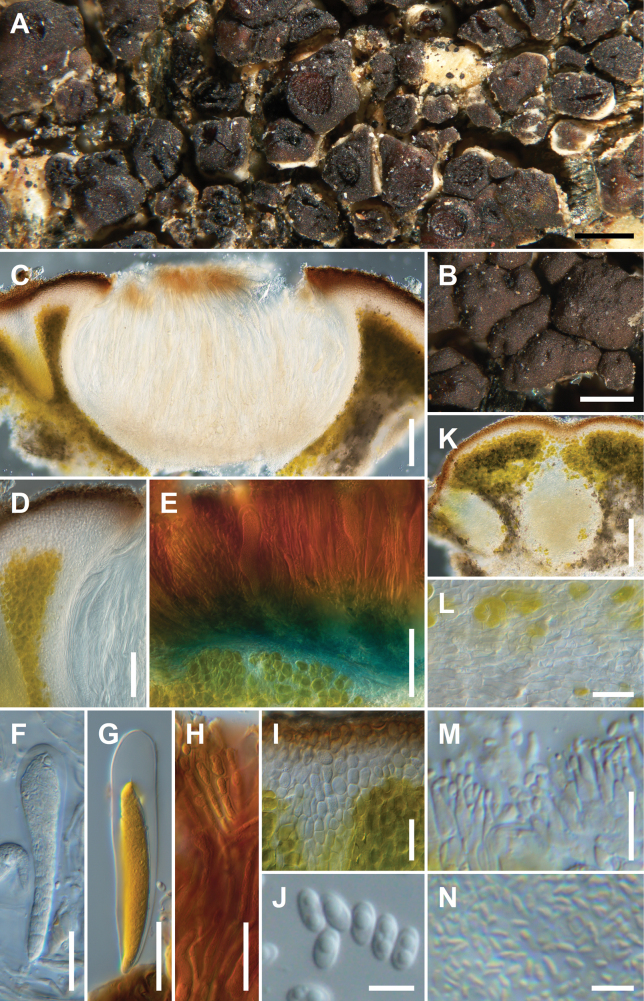
*Trimmatothelopsiscalifornica*, Jason Dart 577, Holotype **A** areolate thallus with apothecia **B** thallus areoles with pycnidia **C** vertical section of apothecium **D** apothecial crown **E** hemiamyloid reaction of hymenium and amyloid reaction of subhymenium and bleeding into hypothecium turning it a lighter blue **F** young ascus **G** young ascus in IKI **H** paraphyses in IKI **I** cortex above interrupted algal layer **J** mostly biguttulate ascospores **K** vertical section of the thallus with pycnidia **L** hyphal tissue below pycnidium **M** long ampulliform conidiogenous cells producing conidia **N** mass of conidia in pycnidial gel. Scale bars: 500 µm (**A, B**); 100 µm (**C**); 50 µm (**D, E, K**); 20 µm (**F–I, L**); 10 µm (**M**); 5 µm (**J, N**).

##### Diagnosis.

Similar to *Trimmatothelopsisoreophila* but differing in having regularly elevated apothecia mostly 0.5 mm wide in a dark brown crown, in having areoles with an elevated mycelial base instead of being squamulose with a stipe, and in having short conidia (1.8–)2.0–2.26–2.5(–2.8) × (0.8–)0.82–0.98–1.1(–1.3) µm, l/b 2.3.

##### Etymology.

The name refers to the region in which the species occurs.

##### Description.

Thallus of dispersed or contiguous bullate to irregular areoles, 0.5–1.0 mm wide and ca 0.4 mm high, replicating by division, forming clusters up to 5 mm wide and 1.5 mm high, broadly attached, but subsquamulose with lobes and becoming elevated on a mycelial base. Upper surface epruinose, light or dark brown to reddish brown, smooth or rough, glossy or dull. Lower surface of lobes white. Epicortex 10–25 µm thick. Cortex 40–60 µm thick, of disarticulated hyphae, mostly cells 3–4 µm, upper layer one cell thick, ends of hyphae in brown pigment caps 5–7 µm wide. Algal layer continuous, 60–100 µm thick, some hyphal bundles interrupting the algal layer but may not be seen in every section, algal cells not dense, the lower layer tending to be uneven, extending below apothecia, algal cells mostly 5–12 µm. Medulla obscured with crystals and gelatinization, hyphae intricate 3–5 µm wide. Apothecia usually 1–6 per areole, 0.2–0.6 mm wide, disc black, rough, epruinose. Margin elevated, slightly higher than the disc, color of thallus. Parathecium 40–70 µm wide, hyphae thin 1.4–2.0 µm wide, IKI-, merging with cortex and pushing it up to form the margin. Hymenium 110–170 µm high, epihymenium 15–20 µm thick, light brown, paraphyses 1.5–2.0 µm wide, apices unexpanded, equator up to 400 µm wide, hymenial gel IKI+ hemiamyloid, blue turning red. Asci 90–140 × 15–22 µm, *Acarospora*-type ascus stain, ascospores (4–)4.5–5.13–5.8(–6.2) × (2.1–)2.3–2.45–2.6(–2.8) µm (n = 20), l/b 2.1. Subhymenium 30–50 µm thick, IKI+ blue, euamyloid. Hypothecium 10–17 µm thick, IKI-. Pycnidia 165–244 × 77–140 µm, multi-chambered, conidiogenous cells 6–14 × 1.5–3.0 µm, conidia (1.8)–2.0–2.26–2.5(–2.8) × (0.8–)0.82–0.98–1.1(–1.3) µm (n = 20), l/b 2.3.

##### Habitat and distribution.

*Trimmatothelopsiscalifornica* is so far only known from California. It occurs from sea level to 2330 m on granite, volcanic rock, and schist, in central and south California (Monterey Co., Santa Cruz Island, Santa Monica Mountains, San Bernardino Mountains, San Jacinto Mountains).

##### Additional specimens examined.

U.S.A. California: Los Angeles Co., Santa Monica Mountains, Malibu State Park, Lost Cabin Trail, 34.0933, -118.7405, alt. 240 m, on volcanic rock, 10 Aug. 2009, K. Knudsen 11731 & T. Sagar (SBBG), Riverside, San Jacinto Mountains, Cedar Springs Trail, southwest-facing slope, 33.6644, -116.5766, alt. 1890 m, on schist in underhang, 13 Aug. 2005, K. Knudsen 3493 (NY, SBBG), Devil’s Slide, sunny open area on west-facing slope, 33.7752, -116.6780, alt. 2330 m, on granite with *Aspicilia* species, 15 Sept. 2006, K. Knudsen 7194 (SBBG), near trail to Round Valley, 33.8075, -116.6522, alt. 2670 m, frequent on granite, 17 Nov. 2006, K. Knudsen 7889 (SBBG); San Bernardino Co., San Bernardino Mountains, pebble plain along Polique Canyon Road, 34.305, -116.85083, alt. 2280 m, on small granite pebbles, 25 Aug. 2010, K. Knudsen 13676.1 & S. Eliason (SBBG); Santa Barbara Co., Santa Cruz Island, High Mount, 34.0314, -119.5824, alt. 410 m, on volcanic rock, 19 July 2012, K. Knudsen 14985 & J. Kocourková (SBBG); Ventura Co., Santa Monica Mountains, Point Mugu State Park, base cliffs above normal high tide level, 34.0986, -119.0763, 3 m, on volcanic rock, 10 Oct. 2005, K. Knudsen 4067.2 & M. Knudsen (SBBG).

##### Notes.

*Trimmatothelopsiscalifornica* differs from other species in the genus in having short conidia. It can easily be confused with *Acarosporaelevata* H. Magn., a species often on granite at high elevations from California to the Rockies (Knudsen 2007). *Acarosporaelevata* has an elevated parathecial crown but has usually a lower hymenium than *T.californica* (ca 60–120 µm) as well as dark blue euamyloid hymenial gel vs. IKI+ hemiamyloid hymenial gel.

Specimens of *Trimmatothelopsis* can be misidentified as *Myriospora*. *Myriospora* differs in having shorter conidia usually less 3 µm long. Both are well-supported as separate genera (Fig. 1; Westberg et al. 2011; Knudsen et al. 2021c). Because of the height of hymenium and a poor understanding of *Myriospora* taxonomy at that time in North American lichenology, *T.californica* was identified as *Acarosporascabrida* Hedl. ex H. Magn. (Knudsen 2005, 2007). The circumscription of *A.scabrida* is heterogenous in Knudsen (2007). We do not recognize *Myriosporascabrida* (Hedl. ex H. Magn.) K. Knudsen & L. Arcadia as occurring in California.

#### ﻿ Trimmatothelopsis mexicana

Taxon classificationFungiAcarosporalesAcarosporaceae

K. Knudsen, Huereca, Kocourk. & Hodková sp. nov.

1BCA3FC7-D9F3-55E7-B2CD-1493777D2310

: MB845676

Fig. 3

##### Diagnosis.

Similar to *Trimmatothelopsisterricola* but differing in producing long conidia (up to 13.9 × 1.5 µm).

##### Type.

Mexico, Nuevo León: Sabinas Hidalgo, Presa Sombretillo, on exposed siliceous boulders at the edge of dam crest, Tamaulipan thorn scrub forest with *Acaciarigidula*, *Cordiaboissieri* and *Prosopisglandulosa*, on red sandstone, 26.3220, -99.9519, alt. 360 m, 28 Dec. 2020, A. Huereca AH-877 (holotype-BRY-C).

**Figure 3.**
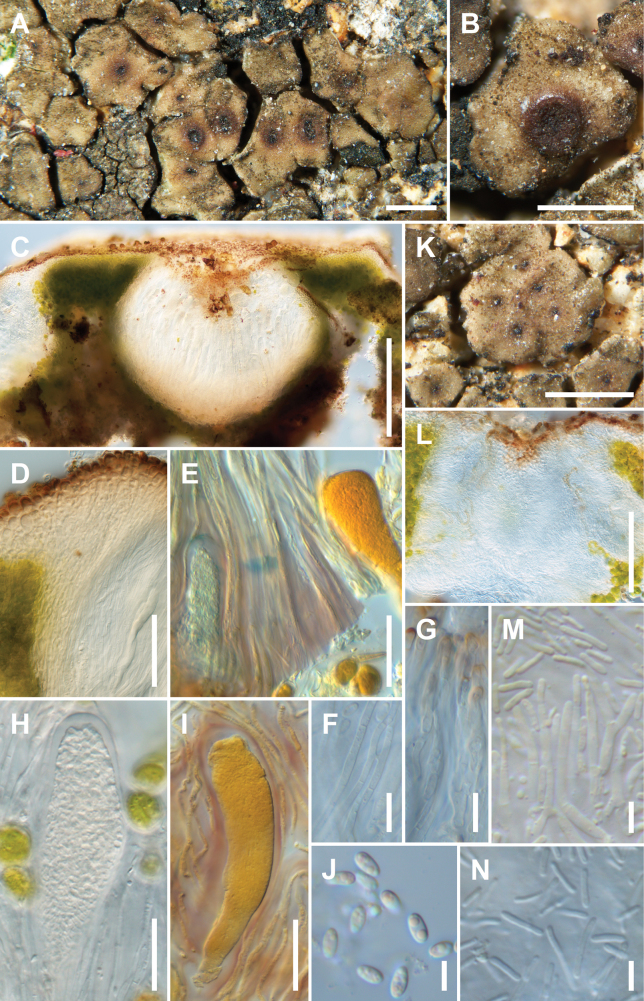
*Trimmatothelopsismexicana*, Huereca AH-877, Holotype **A** areolate thallus with apothecia **B** apothecium with elevated crown **C** vertical section of apothecium **D** apothecial crown **E** hemiamyloid reaction of hymenium and amyloid reaction in tholus **F** sparingly anastomosed paraphyses **G** terminal cells of paraphyses **H** young ascus **I** young ascus in IKI **J** mostly biguttulate ascospores **K** pycnidia on the thallus **L** vertical section of pycnidium with three chambers **M** long narrowly ampulliform conidiogenous cells producing conidia **N** conidia. Scale bars: 500 µm (**A, B, K**); 200 µm (**C**); 100 µm (**L**); 50 µm (**D**); 20 µm (**E, H, I**); 10 µm (**F, G**); 5 µm (**J, M, N**).

##### Etymology.

This is the first species of the genus discovered in Mexico and is named in honor of the work of all the Mexican lichenologists in North America.

##### Description.

Thallus of squamules forming a dispersed to areolate pattern, 0.5–1.2 mm wide, 300–500 μm thick including stipe, replicating by division. Upper surface pale brown, epruinose, lower surface brown and ecorticate. Epicortex 10 μm thick. Cortex 20–30 μm thick, top layer brown, one cell thick, cells up to 7 μm wide, lower layer hyaline, cells mostly round 3–5 μm wide. Algal layer 70–120 μm thick, uninterrupted, dense, algal cells 8–12 μm wide. Medulla 60–100 μm thick, hyphae 0.5–1.0 μm, obscure, upper area hyaline, lower area inspersed and darker, hyphae funneling into the stipe. Apothecia usually one per squamule, sometimes two or three, sometimes with compound apothecia, disc punctiform 1–2 mm wide, rarely 4 mm wide, concave, disc brown and reddish when wetted, epruinose, sometimes with elevated apothecial crown, color of the thallus, often with a red ring around the base of apothecia. Parathecium 10–40 μm wide, merging with cortex, IKI-. Hymenium 200–220 µm high, paraphyses 1.9–2.3 μm wide, apices in brown gel cap, hymenial gel IKI+ red, hemiamyloid. Asci 110–120 × (20–)30–40 μm, ascus stain IKI+ light blue tholus and space between the outer and inner wall of the ascus before ascospores fill the asci, darker blue area in upper layers of tholus evanescent, observed once. Ascospores (3.8–)4.8–5.24–5.7(–5.8) × (2.3–)2.4–2.55–2.8(–3.1) µm (n = 20), l/b 2.1. Subhymenium ca 10–20 μm tall, IKI+ blue, fading and hard to distinguish from hymenium. Hypothecium 20–35 μm tall, IKI-, usually narrowing along the side of hymenium into the parathecium. Pycnidia 70–107 × 128–178 µm, multi-chambered, with conidiogenous cells 21–32 × 1.9–2.8 µm (n = 10), conidia variable (4.1–)5.0–8.2–11.4(–13.9) × (0.9–)1.0–1.18–1.4(–1.5) µm (n = 20), l/b 7.0. Not producing secondary metabolites.

##### Ecology and distribution.

On siliceous red sandstone, known only from the type locality at Nuevo León, Sabinas Hidalgo, Presa Sombretillo, at an altitude of 385 m.

##### Notes.

*Trimmatothelopsismexicana* has the same IKI ascus stain with a light blue tholus and blue space between inner and outer layer of the ascus as in five other species of *Trimmatothelopsis*. The blue stain in upper tholus was observed once. It differs from all species in genus in having the longest conidia and the second longest conidiogenous cells. *Trimmatothelopsisrhizobola* has the longest conidiogenous cells (Knudsen and Lendemer 2016). They are filiform, 15–20(–40) × 1 μm.

We were expecting *Trimmatothelopsismexicana* in the Chihuahuan Desert in New Mexico, but did not find it. Instead we discovered another new species, *T.novomexicana*. *Trimmatothelopsismexicana* is currently known only from the type locality. We are sure someone will collect this distinctive species in Mexico in the future, and it may occur at least in New Mexico or Texas in the United States.

#### ﻿ Trimmatothelopsis novomexicana

Taxon classificationFungiAcarosporalesAcarosporaceae

K. Knudsen, Kocourk. & Hodková sp. nov.

FFB2F3FD-6E80-5BD9-A10B-4FA58B745B0C

: MB845678

Fig. 4

##### Diagnosis.

Similar to *Trimmatothelopsisgordensis* but differs in having a contiguous epilithic areolate thallus instead of a thallus of dispersed areoles and being distinct in nrITS and mtSSU sequence data.

##### Type.

U.S.A., New Mexico: Eddy Co., Brokeoff Mountains Wilderness Study Area, pinyon-juniper woodland, 32.2056, -104.8418, alt. 1850 m, on Permian limestone, 27 Mar. 2022, J. Kocourková 10875 & K. Knudsen (holotype-PRM; isotype-BRY-C).

##### Etymology.

This species is named after the state of New Mexico where it was discovered.

##### Description.

**Figure 4.**
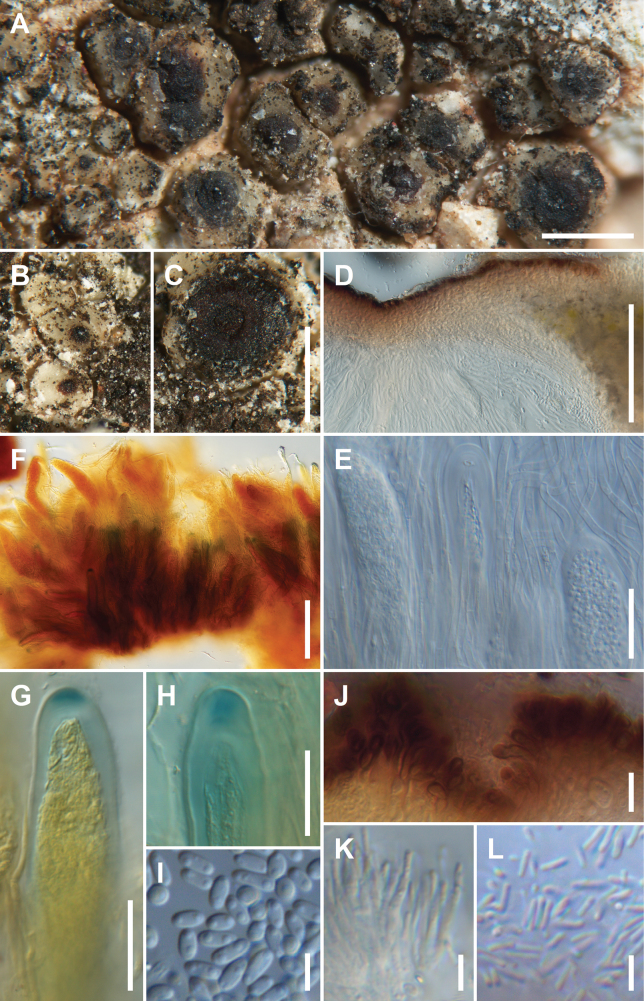
*Trimmatothelopsisnovomexicana*, Kocourková 10875, Holotype (**B–D, F, I**); Knudsen 19324 (**A, E, G, H, J–L**) **A** areolate thallus with apothecia **B** thallus areoles with pycnidia **C** mature apothecium **D** vertical section of apothecium with short parathecial crown **E** young asci and septate paraphyses **F** hemiamyloid reaction of hymenium with amyloid ascus tholus **G** amyloid ascus wall in IKI **I** ascospores **J** ostiolar part of pycnidium with prolongated brown terminal hyphae **K** long narrow ampulliform conidiogenous cells producing conidia **L** conidia. Scale bars: 500 µm (**A–C**); 100 µm (**D, F**); 20 µm (**E, G, H**); 10 µm (**J**); 5 µm (**I, K, L**).

Hypothallus with scattered algal cells. Thallus of contiguous areoles, 0.1–0.5 mm and 0.2–0.6 mm, forming patches up to 3 cm wide, or often on rough rock forming smaller patches ca 3–5 mm wide, replicating by division. Upper surface white from epicortex or brown from cortex, epruinose. Epicortex thick 25–40(–70) µm, with distinct hyphae. Cortex 30–60 µm thick, of vertical hyphae, mostly 1 µm wide, apices slightly expanded to 2 µm, upper layer usually one cell thick and light brown, lower layer hyaline, cortex sometimes completely lacking between algal layer and a thick epicortex. Algal layer of scattered algal cells 2–3(–7) µm wide, sometimes continuous or in small clusters throughout the thallus, sometimes continuous below apothecia but sometimes absent or with only a few scattered algal clusters. Medulla 100–250 µm thick, obscure with substrate crystals, hyphae ca 1–2 µm wide, a few scattered algal cells. Apothecia scattered, disc small usually 100–200 µm µm wide, without a distinct thalline ring, disc black or red, epruinose, immersed, becoming convex. Parathecium indistinct to 10 µm wide. Hymenium 200–300 µm tall, epihymenium reddish, 10 µm thick, paraphyses 1–2 µm wide with unexpanded apices, hymenial gel IKI+ red or light blue turning red, hemiamyloid, but if IKI too diluted with water on the slide the reaction is IKI- pale yellow. Asci 130–160 × 20–35 µm, cylindrical, tholus and space between inner and outer wall of ascus pale blue, with a dark blue stain in the upper layers of tholus, ascospores ellipsoid, (4.4–)4.7–5.31–5.9(–6.5) × (1.7–)1.9–2.21–2.5(–2.8) µm (n = 20), l/b 2.4. Subhymenium 0–40 tall, IKI+ blue (but reaction often negative like hymenium). Pycnidia 80–100 × 130–180 µm, multi-chambered, terminal cells of ostiole hyphae dark brown and 2.7–4.6 µm wide (Fig. 4K), conidiogenous cells 7.5–13.3 × 1.4–2.1 µm (n = 20), conidia (3–)3.5–4.27–5.0(–5.7) × (0.8–)0.8–0.99–1.1(–1.3) µm (n = 20), l/b 4.4. Not producing secondary metabolites.

##### Additional specimens examined.

U.S.A., New Mexico: Eddy Co., Brokeoff Mountains Wilderness Study Area, pinyon-juniper woodland, 32.2056, -104.8418, alt. 1870 m, on Permian limestone, 26 Mar. 2022, J. Kocourková 10877 & 10888 & K. Knudsen (Hb. K&K); Lincoln Co., Carrizozo Malpais, Valley of Fires Recreation Area, at base of the sandstone slope above the lava fields, junipers common, 33.6817, -105.9247, alt. 1950 m, on reconsolidated calcareous sandstone, 24 Mar. 2020, K. Knudsen 19324 & J. Kocourková (SBBG).

##### Ecology and distribution.

The holotype was collected on scattered limestone rocks in full sun on a low crest in the Chihuahuan Desert in New Mexico at an elevation of 1850 m. It was growing in pinyon-juniper woodland on uplifted and eroded Permian reefs. It was associated with *Acarosporapeltastica* and *Circinariacontorta*. We expect it to be locally frequent and to occur in the adjacent Guadalupe National Park in pinyon-juniper areas. It was definitely rare about 100 miles from the type locality at the Carrizozo Malpais lava beds, on calcareous reconsolidated sandstone, growing with Peltulaobscuransvar.deserticola, at the base of a north-facing sandstone slope with junipers at elevation of 1950 m. We studied this area extensively and only collected it once. *Trimmatothelopsisnovomexicana* often occurred in small patches with a few apothecia among other lichens on rough limestone.

##### Notes.

Four other species of *Trimmatothelopsis* have similar ascus stains with a darker blue stain in upper layers of a pale blue tholus: *T.americana*, *T.gordensis*, *T.mexicana* and *T.wirthii*. *Trimmatothelopsisnovomexicana* differs from *T.americana* especially in having apothecia lacking a carbonized outer surface. *Trimmatothelopsisnovomexicana* differs from *T.gordensis* especially in having areolate thallus vs. a thallus of discreet areoles. *Trimmatothelopsisnovomexicana* differs from *T.mexicana* in having longer conidia and in lacking a stipe. *Trimmatothelopsisnovomexicana* differs from *T.wirthii* especially in having areoles vs. large squamules up to 7 mm wide.

The narrow morphological and genetic differences between these similar species are probably based on a long geographical isolation from each other. One mystery of the protologue of *Thelocarpellagordensis* was a description of periphyses in the ostiole of the ascomata (see the drawing in Navarro-Rosinés et al. 1999). Our colleague Martin Westberg borrowed the holotype from Claude Roux. He was told he could photograph it (a photograph we used in this study) but could not dissect any of the areoles (Westberg, pers. comm.) Later, based on its recovery in the Acarosporaceae, the description of periphyses in *gordensis* was treated as a misinterpretation of the incurving melanized hyphae of the parathecium merging with the cortex around the punctiform discs (Knudsen and Lendemer 2016). In studying *Trimmatothelopsisnovomexicana* we discovered the source of the misinterpretation of *T.gordensis* as a perithecioid lichen. The authors had mistaken the elongated black tips of the terminal cells of ostiole hyphae of the pycnidia for being hyphae of a perithecia (see Fig. 4J). In the specimens of *T.novomexicana* there is also no evidence apothecia grow out of stromata that contained pycnidia as in *Sarcogynesimilis* H. Magn. (see pictures of the synonym *Sarcogynereebiae* K. Knudsen in Knudsen et al. 2011). In the recent description of *Trimmatothelopsiswirthii* (see below) it is stated there are no periphyses in the “ostiole”. Roux also describes pycnidia with dark ostiole hyphae. Roux described melanized horizontal hyphae incurving from the parathecium and merged with the cortex around the punctiform apothecia as pseudopapillae (Roux 2021).

#### ﻿ Trimmatothelopsis wirthii

Taxon classificationFungiAcarosporalesAcarosporaceae

(Cl. Roux) K. Knudsen & Kocourk. comb. nov.

83E37E2E-D77B-5580-9525-16D8C405D4BF

: MB845680

= Thelocarpellawirthii Cl. Roux, syn. nov.

##### Type.

France, massif des Vosges, département du Haut–Rhin, Rossberg, Vogelsteine (rochers des oiseaux), alt. 1060 m, sur une face verticale d’un rocher d’andésite, 27. Aug. 2020, V. Wirth, herb. Cl. Roux (holotype, n.v., isotype, SNMS, n.v.), syn. nov.

##### Description.

See Roux (2021). Roux treats *Trimmatothelopsiswirthii* as having a perithecioid ascomata based upon a morphological genus concept we have already rejected (Knudsen and Lendemer 2016). Also see notes below and for *Trimmatothelopsisnovomexicana*.

##### Ecology and distribution.

Known only from the type collection from France massif des Vosges, département du Haut–Rhinn, collected on calcareous andesite on a vertical rock face at 1060 m. The size, shape and pale color is similar to some montane specimens of *T.oreophila* which differs in having an *Acarospora*-type ascus stain.

##### Notes.

*Thelocarpella* is a morphological genus concept which treats two species of Acarosporaceae as perithecioid lichens with periphyses or pseudoparaphyses (Navarro-Rosinés et al. 1999; Roux 2021). We treat these two species as Acarosporaceae with paraphyses. Roux (2021) has accused us of promoting a phylogenetic concept of *Trimmatothelopsis*. Our concept of the genus *Trimmatothelopsis* is integrative, based on the congruence between phylogenetic data and classic taxonomic analysis (Leavitt et al. 2018; Lücking et al. 2021). In the results section we discuss the distribution IKI+ blue ascus in *Trimmatothelopsis* which does not support the idea that the stain distinguishes *Thelocarpella* from all other species in *Trimmatothelopsis*. For further discussion of *T.wirthii* see discussion of *T.novomexicana* above.

Unfortunately, the type of *Trimmatothelopsiswirthii* was not sequenced but it would be in the *Trimmatothelopsis* clade based on its anatomy and morphology. Its large squamules up to 7 mm in width distinguish *Trimmatothelopsiswirthii* from all other species in genus. Though disagreeing with Roux about the genus *Thelocarpella* we praise him for his excellent description of *A.wirthii* despite treating it as a perithecioid lichen.

### ﻿World Key of *Trimmatothelopsis* with taxonomic citations

**Table:** 

1	Ascus stain IKI+ blue	**2**
–	Ascus stain IKI-	**7**
2	Hymenial gel K/I+ blue black, Australia	***Trimmatothelopsismontana*** (McCarthy 2008)
–	Hymenial gel IKI+ hemiamyloid (red or blue changing to red) or sometimes IKI- yellow in *T.novomexicana*	**3**
3	Carbonized apothecia, North America	***T.americana*** (Knudsen et al. 2011 see microscopic pictures; Knudsen and Lendemer 2016)
–	Without a carbonized apothecia	**4**
4	On silicious rock	***T.mexicana*** (this paper)
–	On calcareous rock	**5**
5	Squamulose, France	***T.wirthii*** (Roux 2021)
–	Areolate	**6**
6	France	***T.gordensis*** (Navarro-Rosinés et al. 1999)
–	Chihuahuan Desert, New Mexico	***T.novomexicana*** (this paper)
7	Ascospores globose 7–10(–12) μm, or broadly ellipsoid 7–9 × 5–7 µm, on base and calcareous rock, Asia, Europe, North America	***T.schorica*** (Knudsen et al. 2011, 2021b, 2022)
–	Ascospores ellipsoid, not globose or broadly ellipsoid, less than 7–9 × 5–7 µm	**8**
8	With carbonized apothecia on siliceous rock	**9**
–	Without carbonized apothecia on calcareous and/or non-calcareous rock or in soil crusts	**10**
9	Rare, coastal France	***T.versipellis*** (Gueidan et al. 2014)
–	Rare (?), coastal Korea	***T.cornea*** (Kondratyuk et al. 2015)
10	On calcareous and non-calcareous rock, brown areoles, often with black margin, apothecial disc to 0.7 mm wide, North America	***T.dispersa*** (Knudsen and Lendemer 2016)
–	On non-calcareous rock or in soil crusts	**11**
11	On non-calcareous rock (especially granite)	**12**
–	In soil crusts	**13**
12	Squamules up to 4 mm wide, pale white to dark brown, North America (California, Oregon)	***T.oreophila*** (Knudsen et al. 2021b)
–	Areoles with elevated apothecia in dark brown crown	***T.californica*** (this paper)
13	Rhizohyphae not in bundles, on compacted clay on sea cliffs, endemic to Ireland and U.K.	***T.benedarensis*** (Knudsen et al. 2021b, 2021c)
–	Rhizohyphae in bundles	**14**
14	Rhizohyphal bundles, thick and root-like, 100–200 µm in diam., Greenland to U.K.	***T.rhizobola*** (Knudsen and Lendemer 2016)
–	Rhizohyphal bundles, not thick and root-like, 40–60 µm in diam., western North America	***T.terricola*** (Knudsen and Lendemer 2016)

## ﻿Conclusion

*Trimmatothelopsis* is a fascinating genus whose evolutionary story needs to be explored through phylogenomic analysis which is beyond the scope of this study.

## Supplementary Material

XML Treatment for Trimmatothelopsis californica

XML Treatment for Trimmatothelopsis mexicana

XML Treatment for Trimmatothelopsis novomexicana

XML Treatment for Trimmatothelopsis wirthii

## ﻿Additional information

### Author ORCIDs

Kerry Knudsen https://orcid.org/0000-0001-5419-5729

Jana Kocourková https://orcid.org/0000-0001-5511-9752

Eva Hodková https://orcid.org/0000-0002-8337-3253

Jason Dart https://orcid.org/0000-0002-5656-066X

Alejandro Huereca https://orcid.org/0000-0002-6460-2380

Jiří Malíček https://orcid.org/0000-0002-3119-8967

### Data availability

All of the data that support the findings of this study are available in the main text or Supplementary Information.

## Supplementary materials

Supplementary material 1 A list of sampled specimens included in the molecular phylogeny Data type docx Explanation note Newly produced sequences are shown in bold. https://binary.pensoft.net/file/920386This dataset is made available under the Open Database License (http://opendatacommons.org/licenses/odbl/1.0/). The Open Database License (ODbL) is a license agreement intended to allow users to freely share, modify, and use this Dataset while maintaining this same freedom for others, provided that the original source and author(s) are credited.Kerry Knudsen, Jana Kocourková, Eva Hodková, Jason Dart, Alejandro Huereca, Jiří Malíček
