# Supplementary material for: ﻿Three new species of Trimmatothelopsis (Acarosporales, Acarosporaceae) from southwestern North America
Source: MycoKeys. 2023 Oct 12;99:251–68. doi: 10.3897/mycokeys.99.102965 (PMC10587834; doi:10.3897/mycokeys.99.102965)
Supplement: Supplementary material 1 — A list of sampled specimens included in the molecular phylogeny [file mycokeys-99-251-s001.docx]

**Supll. Table 1** A list of sampled specimens included in the molecular phylogeny. Newly produced sequences are shown in bold.

| **Species** | **Origin** | **Voucher** | **nrITS** | **mtSSU** | **nrLSU** |
| --- | --- | --- | --- | --- | --- |
| *Acarospora brattiae* | U.S.A., California, Flats Solar Farm | Dart 847.1 (SBBG) | ON303959 | ON303850 | ON303964 |
| *Acarospora destructans* | U.S.A., California, Riverside County | Kocourkova 10557 (hb. K&K) | OM522311 | OM522315 | — |
| *Acarospora privigna* | Germany, Saxony-Anhalt, Harz | Kison 4432/3 (hb. K&K) | OK142747 | OK032132 | ON303622 |
| *Myriospora bullata* | Germany, Sachsen-Anhalt | Schifelbein 4763 (hb. K&K) | MZ262727 | MZ262739 | MZ262749 |
| *Myriospora hassei* | U.S.A, California, Los Angeles Co. | Knudsen 707 (SBBG) | MW715698 | MW715737 | MW715726 |
| *Pleopsidium flavum* | Czech Republic, Prague, Divoka Sarka | Malicek (hb. Malicek) | OK142757 | OK032142 | OP497841 |
| *Pycnora sorophora* | Sweden, Härjedalen | Hermansson 7903a (UPS L-111613) | FJ959357 | AY853338 | — |
| *Sarcogyne fallax* | Portugal, Papagovas, Loininha | Zaca 2347 (hb. K&K) | MZ262722 | MZ262734 | MZ262744 |
| *Sarcogyne hypophaea* | Italy, South Tyrol, Vinschgau, Schlanders | Knudsen 18343 (hb. K&K) | OK142760 | OK032145 | ON303621 |
| *Sarcogyne similis* | U.S.A., California | Dart 1332 (hb. K&K) | MW715720 | MW715741 | MW715730 |
| *Timdalia intricata* | Sweden, Härjedalen | M. Westberg (hb. K&K) | ON303957 | ON303848 | ON303962 |
| *Trimmatothelopsis californica* | U.S.A., California, Monterey Co. | Dart 577 (SBBG) | **OP404922** | **OP404919** | **OP497839** |
| *Trimmatothelopsis dispersa* | U.S.A., Ohio | Lendemer 7189 (SBBG) | ON303960 | ON303851 | — |
| *Trimmatothelopsis gordensis* | France, Drôme, Montbrison–sur–Lez | CR25858 (MARSSJ) | KM879338 | KM879332 | — |
| *Trimmatothelopsis gordensis* | France, Vaucluse, col de Gordes | CR22826 (MARSSJ, holotype) | KM879337 | KM879331 | — |
| *Trimmatothelopsis mexicana* | Mexico, Presa, Somoretillo | Huereca AH871 (PRM) | **OK142770** | **OK032155** | **OP497842** |
| *Trimmatothelopsis novomexicana* | U.S.A., New Mexico, Chihuahuan Desert Co. | Knudsen 19392 (SBBG) | **OP162366** | **OP404920** | **OP216684** |
| *Trimmatothelopsis novomexicana* | U.S.A., New Mexico, Brokeoff Mountains | Kocourkova 10875 (PRM) | **OP404923** | **OP404921** | **OP497840** |
| *Trimmatothelopsis oreophila* | U.S.A., California, Riverside Co. | Knudsen 2410 (SBBG) | MK948458 | MK948477 | — |
| *Trimmatothelopsis oreophila* | U.S.A., California | Knudsen 2366 (SBBG) | MK948460 | MK948479 | — |
| *Trimmatothelopsis rhizobola* | Sweden, Lule Lappmark | Westberg 2994 (LD) | EU870640 | EU870692 | LN810868 |
| *Trimmatothelopsis rhizobola* | Sweden, Lule Lappmark | Westberg 3099 (LD) | EU870641 | EU870693 | LN810869 |
| *Trimmatothelopsis schorica* | Czech Republic, Central Bohemia, Lipenec | Kocourkova 8980 (hb. K&K) | ON303958 | ON303849 | ON303963 |
| *Trimmatothelopsis terricola* | U.S.A., California, Los Angeles Co. | Knudsen 11216 & Sagar (S F256012) | LN810806 | LN810931 | — |
| *Trimmatothelopsis terricola* | U.S.A., California, Los Angeles Co. | Knudsen 11216 & Sagar (S F256013) | LN810807 | LN810932 | — |
| *Trimmatothelopsis versipellis* | France, Finistère, Plogoff | CR25921 (MARSSJ) | KM879336 | KM879327 | — |
| *Trimmatothelopsis versipellis* | France, Finistère, Plogoff | CR25922 | KM879335 | KM879328 | — |
